# Subject Matter Expert Insights for Engaging Underrepresented Older Adults with Disabilities in Research

**DOI:** 10.1093/geroni/igaf122.4366

**Published:** 2025-12-31

**Authors:** Elena Remillard, Maurita Harris, Kyra Miller, Wendy Rogers

**Affiliations:** Georgia Institute of Technology, Atlanta, Georgia, United States; Wilfrid Laurier University, Toronto, Ontario, Canada; Georgia Institute of Technology, Atlanta, Georgia, United States; University of Illinois Urbana-Champaign, Champaign, Illinois, United States

## Abstract

The population of older Americans is increasingly diverse. To keep pace with this demographic shift and advance gerontological research, it is critical that research samples reflect this diversity, so findings are generalizable and representative. However, there are barriers to engaging individuals from underrepresented racial/ethnic groups, such as a mistrust of government institutions, historical precedence of unethical research, language barriers, and logistical challenges that often impede participation. There are some general recommendations for expanding diversity of research samples and overcoming these barriers, but there is a need to understand more about best practices for older adults with disabilities, which is a population that can be especially challenging to reach. We interviewed Subject Matter Experts (SMEs) who had direct experience (professional or personal) engaging older adults and/or people with disabilities from underrepresented racial/ethnic groups (e.g., Black/African American, Asian American, Hispanic/Latino American, and Native American). Participants (N = 13) included researchers, clinicians, and advocacy group leaders. The SMEs shared insights on effective strategies for recruitment, outreach, study design, and knowledge dissemination. Their recommendations had key themes including establishing reciprocal community partnerships, involving community members on the research team, offering flexible ways to participate, and carefully considering terminology when referring to disability, race, and ethnicity. These interviews with SMEs provided important insights and actionable solutions to improve the diversity and inclusivity of research samples. Ensuring that participant samples reflect the range of persons who will use a product, technology, intervention, or community program will contribute to successful implementation, ultimately improving quality of life for older adults.

